# Complementary and Alternative Therapies for Inflammatory Diseases 2018

**DOI:** 10.1155/2018/2068724

**Published:** 2018-08-30

**Authors:** Ying-Ju Lin, Yuan Xu, Junji Xu

**Affiliations:** ^1^School of Chinese Medicine, China Medical University, Taichung, Taiwan; ^2^Genetic Center, Department of Medical Research, China Medical University Hospital, Taichung, Taiwan; ^3^National Institute of Allergy and Infectious Diseases, National Institutes of Health, Bethesda, Maryland, USA

Inflammation occurs in response to various cellular stress including infection, irradiation, or chemical or physical injury. It helps us counteract infection, clear out necrotic cells, remove damaging chemicals, and initiate tissue repair. Keeping a delicate balance of inflammation response by eliminating harmful stimulus, while still maintaining self-tolerance to avoid host diseases, is critical for the body's health. Insufficient or chronic inflammation could lead to progressive destruction to the organism. Many diseases have been demonstrated to associate with acute or chronic inflammation, such as rheumatoid arthritis, Alzheimer's disease, depression, Kawasaki disease, and even cancer. However, treatment options for inflammatory diseases have been limited to anti-inflammatory medications (e.g., acetaminophen) or steroid hormones. Meanwhile, complementary and alternative medicine (CAM) provide natural and effective protection for those who suffer from inflammatory diseases.

Here, we highlight several original research articles as well as review articles on complementary and alternative therapies for controlling inflammatory diseases as published in this special issue. Y. Wang et al. exhibited that Kangzhi syrup, a traditional Chinese formula, exerted a considerable antitussive effect in ovalbumin-induced cough variant asthma animal model. N. Ebihara et al. found that quercetin, one of the plant-derived polyphenolic compounds, suppressed nitric oxide production from nasal epithelial cells* in vitro*, implicating that quercetin may attenuate allergic rhinitis, one of the allergic diseases. P. C. Marinho et al. showed that capybara oil, a source of polyunsaturated fatty acids, improved hepatic mitochondrial dysfunction, steatosis, and inflammation in nonalcoholic fatty liver disease animal model. P.-C. Chou and H.-Y. Chu reviewed several findings that showed that acupuncture alone or combined with other treatments may be beneficial to the anti-inflammatory effect on rheumatoid arthritis.

In this special issue, we present 17 papers that address the issue about anti-inflammatory effects of complementary and alternative medicine and provide natural and effective protection for those who suffer from inflammatory diseases.

